# Opening a can of centipedes: new insights into mechanisms of body segmentation

**DOI:** 10.1186/1741-7007-11-116

**Published:** 2013-11-29

**Authors:** Guillaume Valentin, Andrew C Oates

**Affiliations:** 1 MRC National Institute for Medical Research, The Ridgeway, Mill Hill, London NW7 1AA, UK

## Abstract

The search for a common developmental genetic mechanism of body segmentation appears to become more difficult, and more interesting, as new segmented organisms are added to the roster. Recent work in this journal by Brena and Akam on segmentation of the geophilomorph centipede *Strigamia maritima*, an arthropod distantly related to the standard insect models, contains developmental and evolutionary surprises that highlight the importance of a wider sampling of phyla.

See research article: http://www.biomedcentral.com/1741-7007/11/112

## 

The evolution of body axis segmentation is the subject of an historical debate in which the search for homologies has recently focused on the developmental mechanisms underlying segment formation in three taxa: annelids, arthropods and chordates. Originally, in his classical Articulata hypothesis based on morphological traits, Cuvier (1817) proposed that annelids and arthropods shared a common segmented ancestor, whereas the chordates had independently evolved segmentation. With the elucidation of the new animal phylogeny based on ribosomal RNA gene sequences in which Bilateria are divided into three ancient clades - Lophotrochozoa, Ecdysozoa, and Deuterostomia (containing annelids, arthropods, and chordates, respectively) - came the argument from parsimony that because segmented bodies are a minority in each clade, they are most likely independently evolved. Two more arguments further defend this hypothesis. First, there is variety of developmental processes underlying segmentation among the three clades; second, the germ layers that are initially segmented are different: with some exceptions, most chordates and arthropods primarily segment mesoderm and ectoderm, respectively - annelids segment both layers at the same time.

Modern evo-developmental biology has now entered this discussion with findings of homology between segmentally expressed genes giving rise to the hypothesis that the last common ancestor of all three clades, *Urbilateria*, was segmented [1]. A corollary is that segmentation of the body axis must have been lost at several points during evolution [2]. In this issue, Brena and Akam have extended the analysis of segmentation expression dynamics in the centipede *Strigamia maritime*[3]. Their new work raises several fundamental questions about the mechanisms and evolution of segmentation in arthropods, and its similarities to that in chordates.

Before examining the candidate molecular systems that have come to light, it is important to highlight the similarities and differences in how various embryos grow and elongate their body axis (germband), and how morphological segmentation is integrated into this growth mode (Figure 1). One extreme is provided by the long germband insects, with the most famous example being the beloved fruitfly *Drosophila*. In these embryos, segmentation occurs simultaneously along the body axis in the absence of elongation. Others, such as the short germband flour beetle *Tribolium*, exhibit simultaneous segmentation of the head parts, but sequentially segment their bodies in concert with posterior growth at a terminal growth zone [4]. The bee *Apis* shows a remarkable intermediate mode: segments form sequentially in a body that, much like *Drosophila*, does not elongate during the process [5]. Overall, the sequential mode, where elongation and segmentation are tightly coordinated, appears to be the most prevalent across the invertebrates, and is shared with the vertebrates.

**Figure 1:**
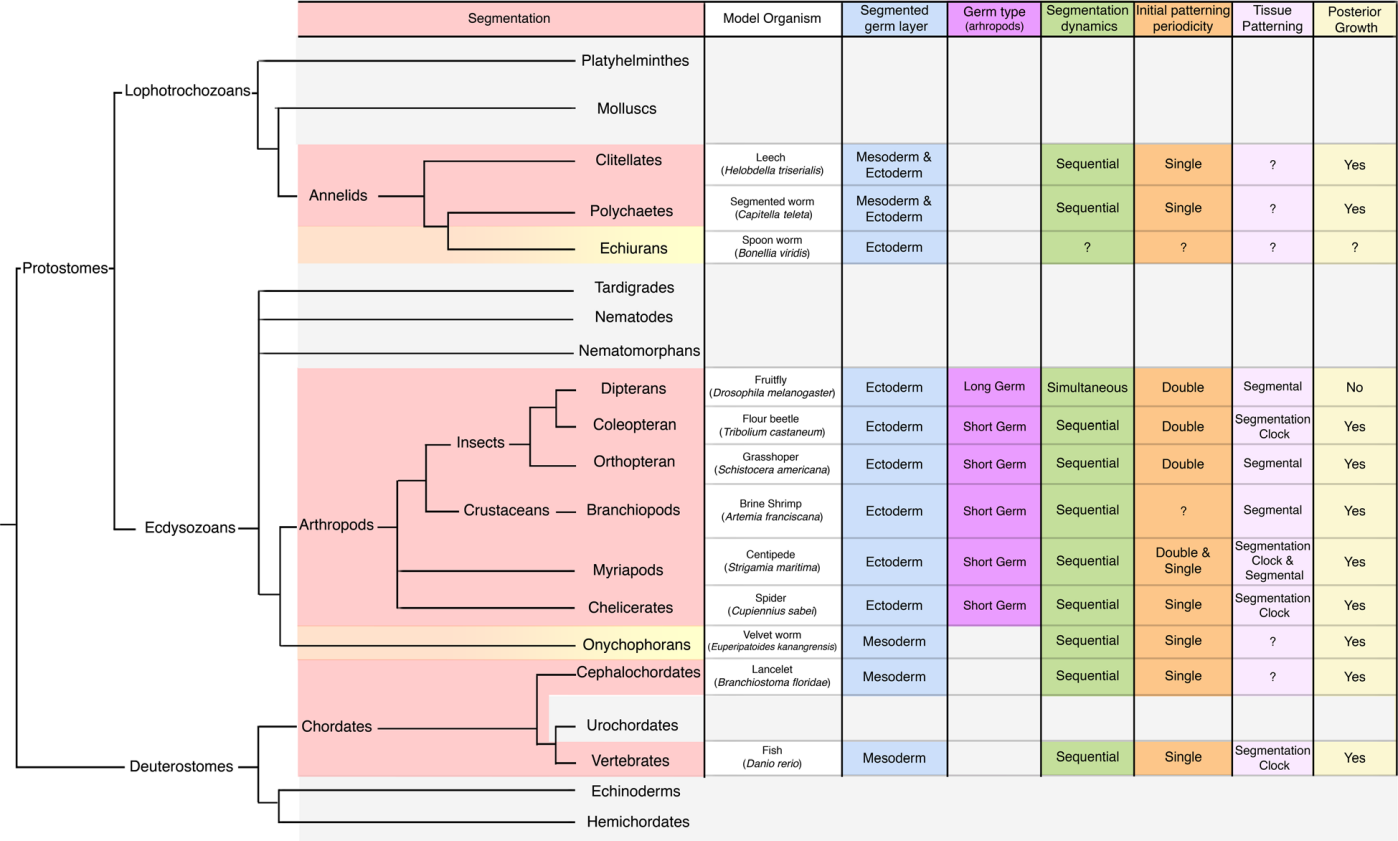
**Bilaterian phylogeny highlighting the key features associated with body segmentation.** For each segmented phylum (red) we listed a representative model organism, the germ layers that are primarily segmented (blue), the germ band development adopted among arthropods (purple), the segmentation dynamics (either sequential or simultaneous addition of segments, green), the initial patterning periodicity (orange), the tissue patterning that underlies segment formation (pink), and whether posterior growth occurs during segmentation (yellow). Onycophorans and Echiurans have a less pronounced, or partial segmentation of the body. The phylogeny is a broad consensus of molecular and morphological traits. Note that we have displayed a trichotomy of pancrustaceans, myriapods and chelicerates, as these relationships are contested. The branch lengths are arbitrary.

What molecular mechanisms underlie these various segmentation systems? In *Drosophila*, the best understood case, maternally supplied signal gradients along the anterior-posterior axis trigger a genetic cascade of transcription factors that subdivide the embryo. This process results in expression of the so-called Pair-rule genes, which initially demarcate a two-segment periodicity [4]: two morphological segments form in the interval along the axis defined by one repeat of pair-rule gene expression. Two-segment periodicity is common in insects, but apart from *Strigamia*, a single segment periodicity is the rule in other arthropods and in vertebrates.

In contrast, evidence from *Tribolium*, cockroach *Periplaneta americana*, and spider *Cupiennius salei*, which all have short germband growth and sequential segmentation, has suggested that a clock-based mechanism is at work across the arthropods [6-8]. Homologs of the *Drosophila* pair-rule gene *Hairy* were among the genes observed with wave-like, cyclic expression patterns in most of these arthropods.

An oscillating molecular mechanism underlying segmentation was first discovered in vertebrates, where a periodic gene expression signal involving *Hairy* gene homologs is converted into regularly sized mesodermal segments, called somites [9]. In this case, each segment along the body axis is formed by the same mechanism, repeating over and over. Therefore, the growth zone in some arthropods and the pre-somitic mesoderm (PSM) in vertebrates can be thought of as a population of genetic oscillators that act as a rhythmic patterning system, or, in other words, a segmentation clock [10]. Strikingly, however, the homology of the oscillating genetic circuits appears weak. The only genes observed with cyclic expression stripes (implying a candidate oscillator component) in any members of both Chordata and Arthropoda are *Hairy* and *Delta* homologs. And yet cyclic expression of these genes is not seen in all arthropods; it is notably absent from the growth zone of *Tribolium*.

Investigating oscillatory or other dynamic genetic processes in species with well-developed sets of molecular and transgenic tools is a formidable challenge. In a species without these tools, or where samples must be collected in the wild, as is the case for *Strigamia*, it is more difficult still. Previous observations of wave-like gene expression patterns, including a *Hairy* homolog, suggested that segmentation in *Strigamia* might be under the control of a segmentation clock. However, without knowledge of the relative movement of cells and dynamics of gene expression, a lineage-based pair-rule mechanism could not be ruled out. In the current paper, Brena and Akam looked in carefully age-matched embryos at the expression of a pair-rule gene, *even-skipped*, and the Notch ligand *Delta*, comparing their wave-like patterns to morphological changes during trunk segmentation (Figure 2). They were able to exclude a prominent contribution of cell movement to the patterns of *eve* or *Delta* gene expression. Furthermore, to demonstrate that these dynamic expression patterns reflect intracellular changes in gene expression they used an intron probe to detect the onset of cyclic gene transcription. Even in the absence of live embryo imaging of cyclic gene expression or explant culture these results converge towards demonstrating the existence of a segmentation clock operating in centipede. Although this conclusion may have been anticipated, three new questions arise from the precise description of segmentation provided in the paper.

**Figure 2.**
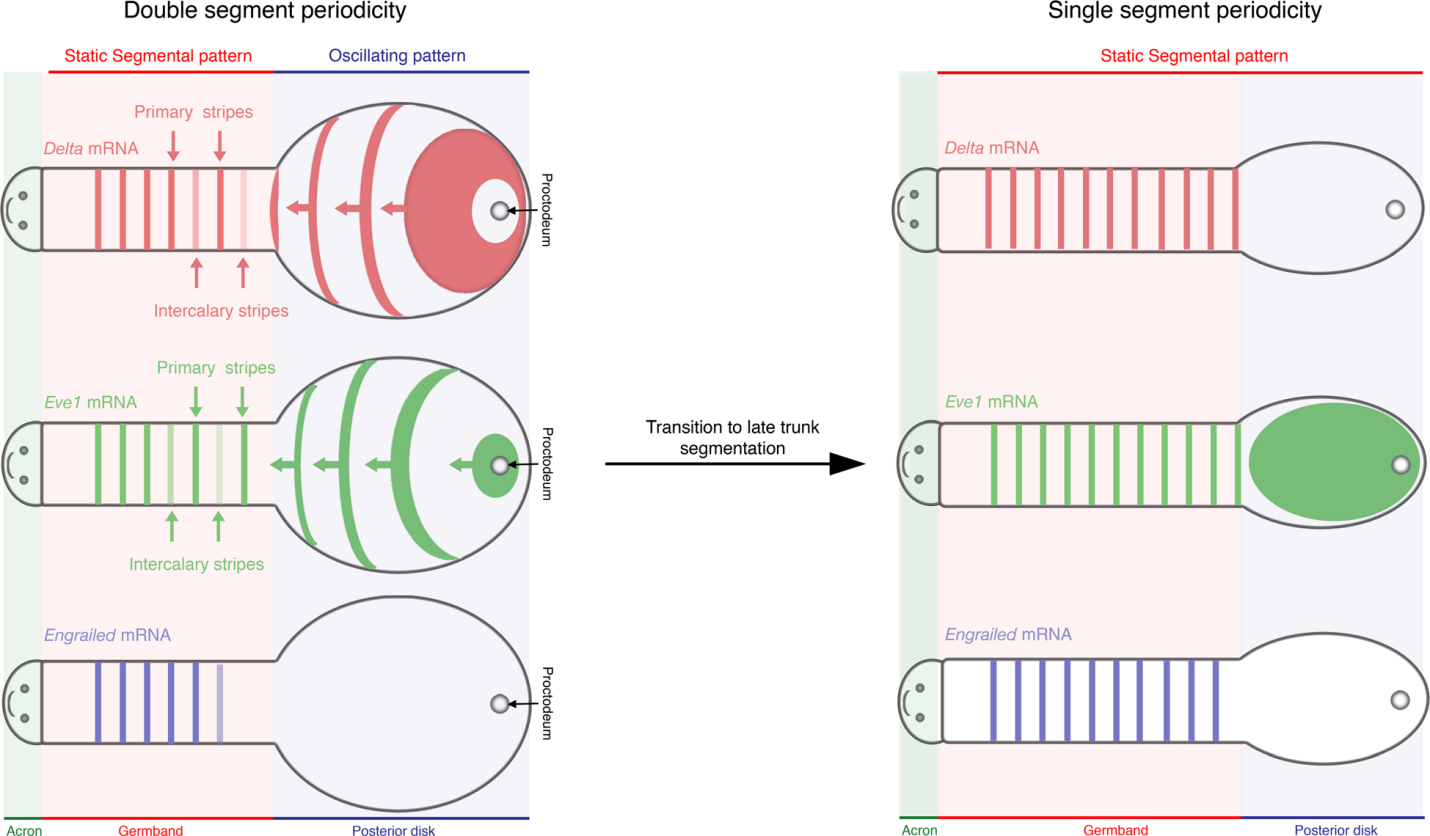
**Graphical representation of *Delta*, *Eve1 *and *Engrailed *expression in *Strigamia*.** Formation of the posterior head and trunk segments is under the control of a clock-like mechanism that manifests as a burst of gene expression in the peri-proctodeal area. *Delta* and *Eve1* expression oscillate out of phase and propagate anteriorly through the posterior disk as a cyclic wave of gene expression. Once the primary stripes of either *Delta* or *Eve1* reach the forming germ-band they stop and *Eve1* and *Delta* intercalary stripes appear. Shortly after, *Engrailed* is expressed in every stripe and a new morphological segment becomes visible. However, when the last nine segments are added to the trunk *Eve1* and *Delta* oscillations cease. At this stage *Eve1* is homogenously expressed in the posterior disc, and a single stripe emerges from this domain in the germ band. *Delta* expression is limited to a stripe that co-localizes with the *Eve1* stripe observed in the germ band. As described for the more anterior segments, *Engrailed* is then expressed and segments are formed.

## Head patterned like the body

In several arthropods, including, for example, *Tribolium*, the head appears to be segmented by a distinct mechanism, as described above for *Drosophila*, which occurs prior to and independently of sequential body segmentation. In *Strigamia*, however, Brena and Akam now show that the segments of the posterior head are demarcated by early expression waves of *Eve* and *Delta* that sweep across most of the blastoderm. These waves appear to be contiguous with those that segment the body, suggesting that the posterior (gnathal) head and body segments are generated by the same mechanism. Given *Strigamia*’s phylogenetic position, this raises the possibility that a clock-like mechanism ancestrally patterned much of the head, and that extant head segmentation modes have been subsequently elaborated from this base. Examination of head segmentation in other basal arthropods, or in members from deeper outgroups, should shed light on this possibility.

## Two-segment to one-segment periodicity within one body

The body of *Strigamia* is generated in phases with two timescales. The first phase is characterized by rapid formation of the first 38 to 40 leg-bearing segments. During this time, *Delta* and *Eve* primary expression stripes establish a double segment periodicity that predates the formation of morphologically defined segments*.* After this, the remaining nine or so segments are added much more slowly. A fascinating observation is the molecular signature of this switching of gears: the genetic network underlying segmentation appears to shift from an oscillatory to a non-oscillatory mode that correlates with the transition from a double to single segment periodicity. At this time, *Even-skipped2* and *Delta* expression are turned off and *Eve1* is expressed broadly across the growth zone. The existence of another oscillating molecular network that acts as a clock during the final phase of segmentation cannot be ruled out, but perhaps this represents the evolutionary acquisition of a novel segmentation strategy in the posterior. Alternatively, given that the short-bodied centipede *Lithobius* appears to make its segments singly, the oscillating double-segment periodic mechanism in *Strigamia* may have been acquired in the trunk on top of an ancestral single segment periodicity mechanism. Regardless of the exact evolutionary transitions involved, *Strigamia* has shown us two new ways to segment a single axis.

## The role of Delta in segmentation

Comparison of the known cyclic genes in various arthropods reveals a curious split. In *Periplaneta*, *Cupiennius* and *Strigamia* expression of both *Delta* and *Hairy* homologs appear to oscillate. However, in *Tribolium* neither *Delta* nor *Hairy* homologs are cyclic; instead, *eve* and *odd* show cyclic patterns [7,11]. In vertebrates, *in vivo* experiments indicate that intercellular coupling via the Notch-Delta signaling system synchronizes oscillations between neighboring cells [12]. This is required to maintain coherent tissue-level stripes of cyclic gene expression, and consequently sharp somite boundaries. Functional evidence in spider and cockroach has shown that inhibition of Notch signaling impairs segmentation (and alters growth) and this has been interpreted as evidence that a Notch-based mechanism is responsible for the oscillations [6,8]. However, an alternative hypothesis is that Delta-Notch signaling is an ancestral mode of coupling cell oscillations during segmentation. In this case, the parts of the internal oscillator might be able to diverge while the coupling mechanism is maintained.

It is interesting to note that long germband insects lack the patterns of gene expression expected from a segmentation clock; the notion that a clock co-evolved with posterior growth has been proposed [13]. In vertebrates, both theoretical work and *in vivo* experiments indicate that intercellular coupling via Delta-Notch signaling confers robustness to the system in the presence of developmental noise [14]. Potential sources include cell proliferation, local cell rearrangement such as migration or convergent-extension, and stochasticity in gene expression. All these processes are inextricably linked to embryonic growth and axis extension. Although there is currently no way to compare gene expression noise between these species, *Strigamia* is a striking example of very strong tissue deformation driving posterior body axis elongation; the posterior progenitor pool occupies the majority of the germband at the onset of segmentation and its cells likely undergo significant mixing during elongation.

The flour beetle *Tribolium* has a much smaller pool of posterior progenitors in which cell division is likely to be a major contributor to elongation; *Delta* does not oscillate and it is not required for proper segmentation even though *Hairy* is expressed in stripes along the body axis of the embryo [11]. In this case, one can ask whether the oscillating cells of *Tribolium* need active synchronization. This question is still open and will no doubt generate a lot of excitement in the segmentation microcosm. However functional analysis of Delta-Notch signaling in other arthropods and in centipede in particular will be needed to understand if the segmentation clock and coupling via Notch-Delta signaling co-evolved and why this function may have been lost in *Tribolium*.

We hypothesize that patterning the growth zone or the PSM via coupled oscillators may be an elegant and robust mechanism to ensure segmental pattern in a tissue where the scale of cellular rearrangements accompanying germband extension would prevent any lineage-based mechanism from working. Thus, a Delta-based mechanism for coupling may be essential in species where a large pool of posterior progenitors is used. Whether this is an ancestral role or not could be investigated by systematically comparing the phylogenetic distribution of cyclic Delta expression with that of the ‘large progenitor pool’ mode of elongation.

## Conclusions

Even without clear homologies, we may nevertheless find common organizing principles of animal segmentation. The use of some form of segmentation clock is clearly one of these. Needing an active way to synchronize cells if they mix significantly during the movements that drive body elongation might be another, and Notch signaling may perform this role.
